# Genetic analysis of the X chromosome in people with Lewy body dementia nominates new risk loci

**DOI:** 10.1038/s41531-024-00649-7

**Published:** 2024-02-20

**Authors:** Ece Bayram, Paolo Reho, Irene Litvan, International LBD Genomics Consortium, Jinhui Ding, J. Raphael Gibbs, Clifton L. Dalgard, Bryan J. Traynor, Sonja W. Scholz, Ruth Chia

**Affiliations:** 1 Department of Neurosciences, Parkinson and Other Movement Disorders Center, University of California San Diego, La Jolla, CA USA; 2 Neurodegenerative Diseases Research Unit, National Institute of Neurological Disorders and Stroke, Bethesda, MD USA; 3 Computational Biology Group, Laboratory of Neurogenetics, National Institute on Aging, Bethesda, MD USA; 4 Department of Anatomy, Physiology & Genetics, Uniformed Services University of the Health Sciences, Bethesda, MD USA; 5 The American Genome Center, Uniformed Services University of the Health Sciences, Bethesda, MD USA; 6 Department of Neurology, Johns Hopkins University Medical Center, Baltimore, MD USA; 7 Neuromuscular Diseases Research Section, National Institute on Aging, Bethesda, MD USA; 8 Therapeutics Development Laboratory, National Center for Advancing Translational Sciences, Rockville, MD USA

**Keywords:** Risk factors, Dementia

## Abstract

Sex influences the prevalence and symptoms of Lewy body dementia (LBD). However, genome-wide association studies typically focus on autosomal variants and exclude sex-specific risk factors. We addressed this gap by performing an X chromosome-wide association study using whole-genome sequence data from 2591 LBD cases and 4391 controls. We identified a significant risk locus within intron 1 of *MAP3K15* (rs141773145, odds ratio = 2.42, 95% confidence interval = 1.65–3.56, *p*-value = 7.0 × 10^−6^) in female LBD cases conditioned for *APOE ε4* dosage. The locus includes an enhancer region that regulates *MAP3K15* expression in ganglionic eminence cells derived from primary cultured neurospheres. Rare variant burden testing showed differential enrichment of missense mutations in *TEX13A* in female LBD cases, that did not reach significance (*p*-value = 1.34 × 10^−4^). These findings support the sex-specific effects of genetic factors and a potential role of Alzheimer’s-related risk for females with LBD.

## Introduction

Lewy body dementia (LBD), including both Parkinson’s disease dementia and dementia with Lewy bodies, is the second most common neurodegenerative dementia in the European and North American populations^1^. Due to population aging and the fast-growing prevalence of Parkinson’s disease, the global number of LBD cases is projected to rise sharply over the next few decades, placing a substantial burden on healthcare. A striking feature of LBD is the differences between females and males. Males have a higher risk for LBD, with an incidence of 7.1 per 100,000 person-years compared to 4.9 for females^2^. These sex differences extend beyond the relative disease occurrence to more fundamental aspects of the disease. Neocortical Lewy body pathology, which increases the likelihood of LBD phenotype, is more common in males^3^. Males are more likely to have pure Lewy body pathology; females are more likely to have mixed pathologies^4^. Even when females have similar levels of Lewy body pathology as males, they are less likely to manifest the symptoms of LBD^5^, suggesting a differential resilience to the pathology.

The etiology of these sex differences is unknown, but genetic factors are likely to be involved. Several genome-wide association studies (GWAS) in LBD highlighted the role of autosomal genes^6,7^. *APOE*, *GBA*, and *SNCA* variants have been replicated across studies; *TMEM175* and *BIN1* were recently identified in our cohort^6,7^. More importantly, a recent sex-stratified meta-analysis revealed differences: signals in the *GBA* and *SNCA* genes were driven by males^8^, with little or no association signals observed in females at these loci.

Why is it essential to determine the drivers underlying the male-female heterogeneity of LBD? These characteristics may reflect differences in causation and the pathways involved in the underlying pathogenic processes; understanding them opens up new avenues to intervene therapeutically. With a length of 155 megabases (Mb), the X chromosomes account for approximately 5% of the human genome. X chromosomes have reduced genetic diversity and lower mutation rates than autosomal chromosomes^9^, increasing their discovery power in analyses. This effect is amplified by the lower recombination rate at the X chromosome compared to the autosomes^10^, giving rise to longer linkage disequilibrium blocks^11^.

Despite this, genetic association studies routinely exclude the X chromosomes, leading the field to miss critical genetic drivers. This limited focus on autosomal chromosomes is primarily due to the complex quality control measures required to analyze X-chromosomal genetic data, stemming from its unique inheritance pattern and X chromosome inactivation in females^12,13^. Recombination of the X chromosome differs for females and males; females have two X chromosomes similar to autosomal chromosomes, and males have one X chromosome with recombination limited to only the pseudo-autosomal regions (PAR; PAR1 and PAR2) in the X and Y chromosomes^14^. Recently, a pipeline simplifying X chromosome-wide association studies (XWAS) was published^12,13^, and this XWAS approach has already identified new risk loci in Alzheimer’s and Parkinson’s diseases^15,16^.

Here, we evaluated the role of the X chromosome in driving LBD risk by performing an XWAS based on whole-genome sequence data generated for a large cohort of cases diagnosed with LBD and healthy individuals^7^. We first performed XWAS on sex-stratified cohorts, followed by a joint analysis of all samples. As there are indications that the *APOE ε4* allele, a major risk factor for LBD, may operate differently in males and females^8^, we also conducted an *APOE ε4*-conditional analysis to detect independent risk variants. Finally, we performed gene burden testing to identify genes encoded on the X chromosome that may be implicated in the disease process.

## Results

### Single-variant associations and enrichment of enhancers linked to disease risk

Following quality control, whole-genome sequence data from 2591 individuals diagnosed with LBD and 4023 neurologically healthy individuals were available for the study of the X chromosome. A total of 257,854 variants encoded on the X chromosome with a minor allele frequency (MAF) ≥1% were tested for association. We estimated the number of haplotype blocks on the X chromosome to be 6296 in the LBD cohort (Supplementary Figure). Based on this, the Bonferroni threshold for significance was set to 7.94 × 10^−6^ (=0.05/6296), as is the standard for XWAS^17^.

**Fig. 1:**
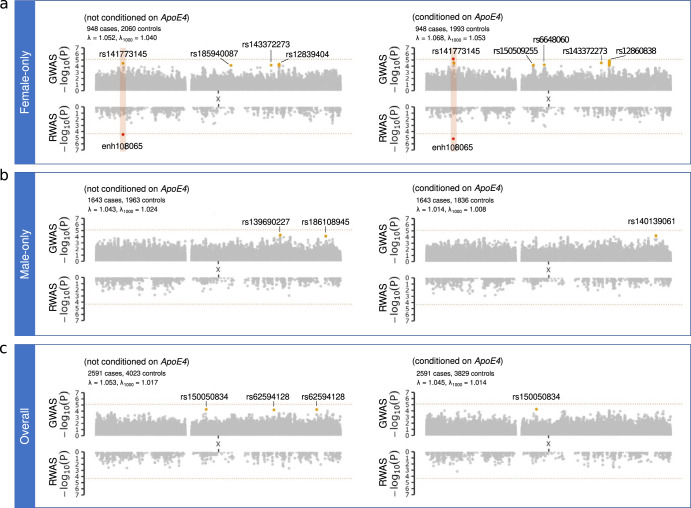
LBD XWAS and RWAS results in female and male LBD case-control cohorts. Panel **a** shows the results for the female-only cohort, Panel **b** shows the male-only cohort, and Panel **c** shows the results for the overall analysis. In each panel, the unconditioned *APOE ε4* results are shown on the left, and *APOE ε4*-conditional results are shown on the right. The x-axis for each plot illustrates the absolute physical position of variants on the X chromosome, and the y-axis denotes the -log10 of the *p*-value. The horizontal dashed line represents the significance level after correction for multiple testing (*p*-value < 7.94 × 10^−6^ for XWAS, and *p*-value < 4.46 × 10^−5^ for RWAS). The number of cases and controls for each analysis and the corrected genomic inflation factor (λ_1000_) for variants with MAF >0.1% are given in each panel. A red dot highlights significant variants or mapped enhancers, and the variants with *p*-values that are one log-fold lower than the significant threshold are depicted by yellow dots (*p*-value < 7.94 × 10^−5^ for XWAS and *p*-value < 4.46 × 10^−4^ for RWAS). The orange-shaded rectangle box shows the overlapping regions for the identified significant hits in the XWAS and the RWAS.

In the female-stratified analysis, a significant association at Xp22.12, located within the intron 1 of the *MAP3K15* gene (rs141773145, odds ratio (OR) = 2.42, 95% confidence interval (CI) = 1.65–3.56, *p*-value = 7.0 × 10^−6^, Table 1, Fig. 1a), was identified when conditioning on *APOE ε4* dosage. Enhancer enrichment analysis maps and significantly associates this risk locus to the promoter GH0XJ019514 (chrX:19513600–19516200), which is specific to the ganglionic eminence-derived primary cultured neurospheres (enh108065, *p*-value = 6.7 × 10^−6^). *MAP3K15* was nominated as the top candidate gene regulated by this enhancer (GeneHancer total score = 200.54, score ranges from 0.01 to 500^18^). Interestingly, the same enhancer was also identified as significantly associated with risk at this locus in the regulome-wide association study (RWAS) (*p*-value = 3.4 × 10^−5^, Fig. 1a).

**Table 1:** Association results for LBD XWAS for sex-stratified and overall analysis

Position	rs-ID	EA	OA	Sex	*APOE ε4* allele condition	OR (95% CI)	*p*-value	EAF Cases	EAF Controls	EAF gnomAD
19,513,849	rs141773145	A	G	Female-only	Not conditioned	2.19 (1.51–3.18)	0.000034	0.0322	0.0153	0.0223
					Conditioned	2.42 (1.65–3.56)	**0.000007**	0.0322	0.0156	0.0223
19,821,829	rs138781955	C	T	Female-only	Conditioned	2.24 (1.54–3.26)	0.000027	0.0322	0.0158	0.0227
19,880,339	rs146313766	G	A	Female-only	Conditioned	2.51 (1.62–3.88)	0.000035	0.0258	0.0113	0.0173
69,593,987	rs140489573	C	T	Female-only	Conditioned	1.77 (1.34–2.35)	0.000075	0.0549	0.0346	0.0470
69,611,258	rs144308131	T	C	Female-only	Conditioned	1.77 (1.34–2.35)	0.000075	0.0549	0.0346	0.0466
69,731,528	rs150509255	A	G	Female-only	Conditioned	1.75 (1.33–2.30)	0.000066	0.0591	0.0374	0.0487
71,648,911	rs150050834	T	C	Joint female & male	Not conditioned	1.40 (1.19–1.65)	0.000052	0.0417	0.0271	0.0316
					Conditioned	1.42 (1.20–1.69)	0.000056	0.0417	0.0270	0.0316
76,575,769	rs6648060	G	C	Female-only	Conditioned	0.47 (0.33–0.68)	0.000061	0.0222	0.0424	0.0356
87,154,921	rs185940087	T	C	Female-only	Not conditioned	2.63 (1.63–4.23)	0.000075	0.0206	0.0085	0.0130
112,297,044	rs143372273	G	T	Female-only	Not conditioned	1.40 (1.18–1.65)	0.000068	0.1503	0.1136	0.1238
					Conditioned	1.45 (1.22–1.72)	0.000029	0.1503	0.1129	0.1238
114,075,787	rs62594128	A	G	Joint female & male	Not conditioned	0.82 (0.75–0.91)	0.000061	0.0913	0.1182	0.1060
117,271,703	rs2840719	T	C	Female-only	Conditioned	1.35 (1.17–1.56)	0.000051	0.2358	0.1869	0.2049
117,341,264	rs10482533	C	T	Female-only	Conditioned	1.32 (1.15–1.51)	0.000046	0.2848	0.2411	0.2450
117,342,494	rs2108104	T	C	Female-only	Not conditioned	1.30 (1.14–1.47)	0.000067	0.2795	0.2316	0.2387
					Conditioned	1.34 (1.17–1.53)	0.000019	0.2795	0.2326	0.2387
117,343,445	rs12394878	A	C	Female-only	Not conditioned	1.30 (1.14–1.47)	0.000072	0.2795	0.2318	0.2384
					Conditioned	1.34 (1.17–1.53)	0.000020	0.2795	0.2328	0.2384
117,344,912	rs12856803	A	C	Female-only	Conditioned	1.33 (1.17–1.52)	0.000025	0.2790	0.2328	0.2389
117,345,435	rs5958254	A	G	Female-only	Conditioned	1.32 (1.16–1.51)	0.000040	0.2753	0.2293	0.2373
117,349,649	rs7052570	C	G	Female-only	Conditioned	1.33 (1.17–1.52)	0.000025	0.2790	0.2328	0.2388
117,351,326	rs12834288	T	C	Female-only	Conditioned	1.33 (1.16–1.52)	0.000027	0.2790	0.2333	0.2391
117,353,885	rs12858772	T	C	Female-only	Conditioned	1.33 (1.16–1.52)	0.000027	0.2790	0.2333	0.2391
117,359,899	rs5956567	G	C	Female-only	Conditioned	1.33 (1.16–1.52)	0.000035	0.2758	0.2293	0.2373
117,363,659	rs2214854	T	C	Female-only	Conditioned	1.29 (1.14–1.47)	0.000070	0.3339	0.2865	0.2947
117,366,012	rs5958292	G	A	Female-only	Not conditioned	1.30 (1.14–1.47)	0.000075	0.2780	0.2299	0.2385
					Conditioned	1.34 (1.17–1.53)	0.000021	0.2780	0.2311	0.2385
117,367,864	rs60964291	A	G	Female-only	Conditioned	1.33 (1.16–1.52)	0.000031	0.2743	0.2270	0.2361
117,369,560	rs2190283	C	T	Female-only	Not conditioned	1.30 (1.14–1.47)	0.000078	0.2774	0.2296	0.2385
					Conditioned	1.34 (1.17–1.53)	0.000021	0.2774	0.2308	0.2385
117,373,825	rs12860838	T	A	Female-only	Not conditioned	1.31 (1.15–1.48)	0.000052	0.2732	0.2194	0.2362
					Conditioned	1.35 (1.18–1.54)	0.000014	0.2732	0.2205	0.2362
117,379,801	rs5958332	A	G	Female-only	Conditioned	1.34 (1.17–1.53)	0.000024	0.2769	0.2301	0.2379
117,382,003	rs6645724	A	G	Female-only	Conditioned	1.33 (1.17–1.52)	0.000027	0.2774	0.2308	0.2384
117,382,755	rs5958342	G	A	Female-only	Not conditioned	1.30 (1.14–1.48)	0.000072	0.2727	0.2211	0.2356
					Conditioned	1.34 (1.17–1.53)	0.000020	0.2727	0.2220	0.2356
117,396,820	rs12839404	A	G	Female-only	Not conditioned	1.31 (1.15–1.49)	0.000048	0.2737	0.2216	0.2340
					Conditioned	1.34 (1.17–1.53)	0.000021	0.2737	0.2223	0.2340
117,398,160	rs79936813	C	T	Female-only	Not conditioned	1.31 (1.15–1.49)	0.000053	0.2727	0.2209	0.2339
					Conditioned	1.34 (1.17–1.53)	0.000022	0.2727	0.2215	0.2339
117,404,930	rs6646880	A	G	Female-only	Not conditioned	1.30 (1.15–1.48)	0.000057	0.2780	0.2308	0.2359
					Conditioned	1.33 (1.16–1.52)	0.000030	0.2780	0.2316	0.2359
118,036,434	rs192452649	T	C	Male-only	Not conditioned	0.84 (0.78–0.92)	0.000058	0.1865	0.2441	0.2043
118,071,219	rs139690227	A	G	Male-only	Not conditioned	0.84 (0.77–0.92)	0.000053	0.1832	0.2401	0.1991
140,922,346	rs7892288	A	G	Joint female & male	Not conditioned	0.83 (0.76–0.91)	0.000054	0.1065	0.1305	0.1290
146,520,931	rs140139061	A	G	Male-only	Conditioned	1.81 (1.36–2.42)	0.000061	0.0280	0.0093	0.0130
146,580,137	rs186108945	T	C	Male-only	Not conditioned	1.66 (1.29–2.13)	0.000074	0.0310	0.0127	0.0152
					Conditioned	1.69 (1.31–2.19)	0.000064	0.0310	0.0131	0.0152

Positions on the X chromosome are shown according to genome assembly hg38. Significant *p*-values are bolded.

*CI* confidence interval, *EA* effect allele, *EAF* effect allele frequency, *gnomAD* genome aggregation database v3.1.2, *OA* other allele, *OR* odds ratio.

There were no significant associations between individual X-linked common variants and LBD risk in the overall or the male-stratified analyses (Table 1, Fig. 1b, c). Similarly, the loci previously reported to be significant in XWASes of late-onset Alzheimer’s disease^16^ and Parkinson’s disease^15^ were not associated with an increased risk of LBD (Table 2).

**Table 2:** LBD XWAS results of loci implicated in Alzheimer’s and Parkinson’s diseases

Position	rs-ID	Nearest gene	OA	EA	Sex	OR	Beta	SE	*p*-value
13,874,463	rs7066890	*GPM6B*	T	C	Female-only	1.00	0.00	0.08	0.97
					Male-only	1.00	0.00	0.05	0.94
					Joint female & male	1.01	0.01	0.04	0.77
85,664,577	rs147122766	Premature ovarian failure critical region	T	A	Female-only	0.95	−0.05	0.07	0.45
					Male-only	0.94	−0.07	0.04	0.12
					Joint female & male	0.94	−0.06	0.04	0.10
126,190,200	rs112930037	*DCAF12L2*	G	A	Female-only	1.05	0.05	0.08	0.54
					Male-only	1.04	0.04	0.05	0.41
					Joint female & male	1.04	0.04	0.04	0.33
154,405,192	rs28602900	*RPL10*	A	G	Female-only	1.00	0.00	0.09	0.98
					Male-only	1.05	0.05	0.05	0.36
					Joint female & male	1.05	0.05	0.05	0.31

LBD XWAS analyses at loci previously implicated in Alzheimer’s disease and Parkinson’s disease identified no significant associations.

*EA* effect allele, *OA* other allele, *OR* odds ratio, *SE* standard error.

Based on the expression quantitative trait loci (eQTL) association summary statistics from Genotype-Tissue Expression Project (GTEx, version 8, https://gtexportal.org/) from the thirteen brain tissue types, none of the index variants in Table 1 were associated with differential gene expression. Furthermore, based on the colocalization analysis, no posterior probabilities of the hypothesis that both traits are associated and share a single causal variant were identified as ≥0.70. None of the index or 1Mb surrounding variants mediated LBD risk through differential expression of any of the genes in the region.

### Rare variant burden testing

**Fig. 2:**
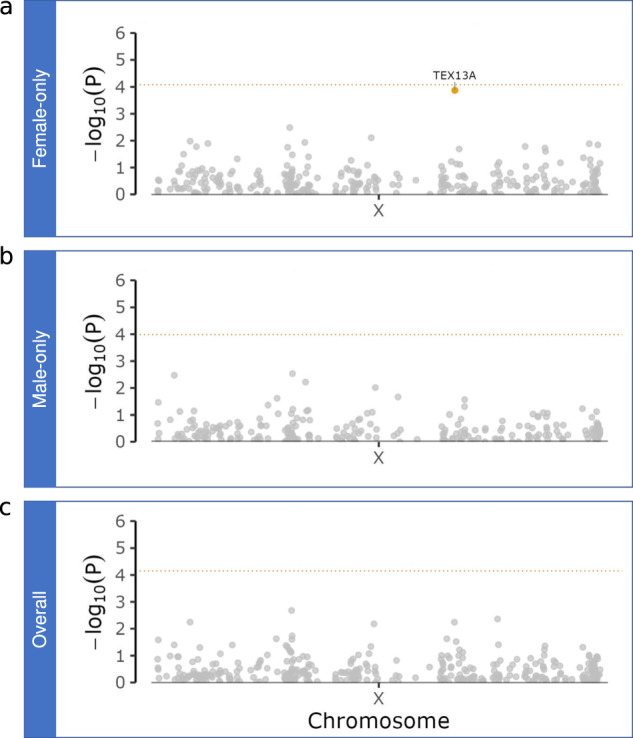
Gene-based rare variant analysis results on X chromosome. Panel **a** illustrates the SKAT results of missense mutations for only females, Panel **b** shows the results for only males, and Panel **c** shows the results in the overall LBD case-control cohort. The dotted, horizontal line indicates the Bonferroni threshold for X chromosome-wide significance (0.05/602 genes = 8.31 × 10^−5^). Genes with *p*-values that are one log-fold lower than the significant threshold are depicted by yellow dots.

To explore whether rare variants within genes contribute to the risk of developing LBD, we performed gene-level sequence kernel association tests (SKAT) of missense mutations with an MAF ≤5% and a minor allele count (MAC) of ≥3 across the X chromosome. This rare variant analysis identified differential enrichment of missense mutations in *TEX13A* among females with *p* value one log-fold lower than the significant threshold (*p*-value = 1.34 × 10^−4^) (Fig. 2a, Tables 3 and 4). One missense variant in *TEX13A* (rs41312550, p.Glu179Val, also an intronic variant for *IL1RAPL2*) was twice as frequent in female cases than in female controls. In contrast, *TEX13A* was not significantly associated with disease in the male-only gene burden analysis (*p*-value = 0.50).

**Table 3:** Rare variant burden testing results

Gene	Number of variants	*p*-value
Female-only analysis		
*TEX13A*	4	1.34 × 10^−4^
Male-only analysis		
*TEX13A*	3	0.51
Joint male & female analysis		
*TEX13A*	4	5.74 × 10^−3^

LBD rare missense variants burden test (SKAT) showed associations that were one log-fold lower than the significant threshold, driven by the female-only study cohort. Range according to hg38 is X:105218928–105220694, X:105218928–105220694.

**Table 4:** Rare missense variants in *TEX13A*

Position	rs-ID	EA	OA	OR	Beta	SE	*p*-value	EAF Cases	EAF Conts	EAF gnomAD	EAC Cases	EAC Conts	Amino Acid Change
105,219,038	rs202235111	C	T	1.08	0.07	1.24	0.9523	0.00053	0.00049	0.00076	1	2	p.N386D
105,219,161	rs377104623	G	A	0.57	−0.56	1.17	0.6325	0.00053	0.00073	0.00066	1	3	p.S345P
105,219,658	rs41312550	A	T	1.99	0.69	0.23	0.0025	0.02110	0.01117	0.01182	40	46	p.E179V
105,219,976	rs41300159	C	T	0.39	−0.95	0.33	0.0046	0.00580	0.01481	0.01298	11	61	p.K141R

Positions are shown according to build hg38. The amino acid change is based on transcript NM_001291277. *P*-value for each variant was derived from the logistic region analysis using Firth’s logistic regression in PLINK2, adjusting for the same set of covariates as described for the common variant analysis.

*Conts* controls, *EA* effect allele, *EAC* effect allele count, *EAF* effect allele frequency, *gnomAD* genome aggregation database v3.1.2, *EAC* effect allele counts, *OA* other allele, *OR* odds ratio, *SE* standard error.

## Discussion

In this XWAS of LBD, our analyses highlight the contributions of the X chromosome variants to the complex genetic architecture of this common but understudied neurodegenerative disease. Specifically, we found that *MAP3K15* is associated with females' risk of developing LBD. In addition, the gene-based aggregation tests implicated mutations in *TEX13A* as playing a role in the pathogenesis of the disease, again among females. The fact that our significant results were only observed in females and not males underscores the sex differences inherent to LBD. These results contribute to the growing efforts to unravel the genetic architecture of LBD^7,19^. Including the X chromosome will likely strengthen future efforts on developing polygenic risk scores and multimodal predictive modeling for the disease, that includes combinations of genetic factors, biomarkers, neuroimaging and clinical data.

We identified a significant association signal within the *MAP3K15* gene that encodes a member of the mitogen-activated protein kinase (MAPK) pathway family. This gene family regulates various cellular activities, including proliferation, differentiation, survival, and apoptosis^20^; disruption of the MAPK pathways has been implicated in other neurodegenerative diseases, such as Alzheimer’s and Parkinson’s diseases^20^. It may contribute to the pathogenesis through the regulation of neuronal apoptosis, β-secretase and γ-secretase activity, and the phosphorylation of tau and amyloid precursor protein; and through neuroinflammatory responses and neuronal death triggered by alpha-synuclein aggregates^20^. However, the MAPK signaling pathway is complex and the functions of the pathway cannot be generalized to a single gene and protein. MAP3K15 belongs to the large MAP3K family, including over 24 characterized proteins, which remains understudied^21^. There is a need to better understand the functional implications of *MAP3K15* variants.

Although the *MAP3K15* gene has not been reported to be associated with neurodegenerative disorders, it has recently been described in a large-scale type 2 diabetes analysis based on a cohort of 454,787 participants from the UK^22^. Protein truncating variants in the *MAP3K15* were associated with a lower risk of diabetes^23^. Diabetes is considered a risk factor for Alzheimer’s and Parkinson’s diseases^24,25^, though its association with LBD is unclear^26,27^. The effect of diabetes on cognitive decline can be sex-dependent, with prediabetes suggested to impair cognition through altering brain metabolism and females being more vulnerable to this deteriorating effect^28^. However, studies suggest no genetic association between diabetes and Alzheimer’s disease^29^, and a negative correlation between diabetes and dementia with Lewy bodies only for males^8^. *MAP3K15* association with LBD risk in females is likely independent of the diabetes association.

Interestingly, we detected the *MAP3K15* gene only in our *APOE ε4*-conditional analysis among females. Mouse models suggest that the MAPK pathways mediate the Alzheimer’s-related pathological effects of *APOE ε4*, including amyloid beta accumulation, tau hyperphosphorylation, synaptic impairments, and reduced vascular endothelial growth factor levels in the hippocampus^30^. Furthermore, γ-secretase activity, amyloid precursor protein and tau phosphorylation associated with Alzheimer’s disease may be sex-dependent, with more severe pathology in females^31,32^. In LBD, Alzheimer’s co-pathology can be more prevalent and impactful for cognitive changes in females than males^33^. Immune response to Alzheimer’s pathology can differ by sex, with potentially more microglial activation in females^34^. Transcriptomics studies in Parkinson’s also support sex differences for inflammation, mitochondrial dysfunction, and oxidative stress. Females show alterations in acidification, microtubule stability, mitochondrial and lysosomal dysfunction, glutamic metabolism and neurotoxicity, whereas males show alterations in pathways related to oxidative stress, inflammation, and innate immune response^35^. Taken together, the sex-specific association of the *MAP3K15* gene observed in our cohort of people with LBD may be related to more prevalent and more severe Alzheimer’s co-pathology in females, coupled with sex-specific effects of genetic factors related to inflammation and oxidative stress.

The potential effect of *MAP3K15* on LBD risk can also expand beyond AD-related mechanisms in females. Our RWAS marked a role for ganglionic eminence in females with LBD. Ganglionic eminences are temporary subcortical gray matter structures that give rise to the basal ganglia, thalamic, olfactory, and the vast majority of cortical interneurons^36^. Dysfunction of the basal forebrain cholinergic neurons and γ-aminobutyric acid (GABA) interneurons stemming from medial ganglionic eminence have been implicated in learning and memory impairment^37^. In Alzheimer’s, Parkinson’s and LBD, the number and density of basal forebrain cholinergic neurons can decline^38^. This decline can be more pronounced in Parkinson’s disease dementia than in Parkinson’s disease, and in dementia with Lewy bodies than in Alzheimer’s disease. Our findings implicate that early developmental factors could mediate an individual’s risk of developing LBD decades later, and there may be a sex difference in this developmental influence.

Rare variant burden testing revealed a differential enrichment of missense mutations in *TEX13A* (rs41312550) in LBD cases compared to controls in the female-only cohort. This finding did not reach significance but had a *p*-value that is one log-fold lower than the significant threshold. *TEX13A*, encoding testis-expressed protein 13A protein, is associated with the degradation of mRNAs encoding particular structural components of elongated spermatids^39^. Interestingly, despite the apparent difference in function and morphology, there are more similarities in the transcriptomic and proteomic profile between the brain and testis than previously appreciated, a pattern shared across mammals^40^. The TEX13A protein was shown to interact with several members of the CCR4-NOT transcription complex family, many of which have been implicated in neurodevelopmental conditions^41,42^. The gene is also differentially expressed in the occipital visual cortex for males and females with Alzheimer’s disease, a part of the brain frequently affected in patients with LBD^43^. These observations may explain how this gene might drive sex differences in LBD. The association can also be due to the *IL1RAPL2* intronic variant and not *TEX13A*. Therefore, this finding should be interpreted cautiously due to the uncertainty.

A limitation of our study is the lack of a replication cohort. A possible alternative was to split the initial cohort into two subsets so that a replication analysis may be possible. However, this approach is impractical for sex chromosomal analysis, as the hemizygosity in males already curtails the effective cohort size. We used different covariates in female and male-only analyses. This is associated with incomplete X chromosome inactivation in females, with up to one-third of genes getting expressed from both the inactive and active X chromosomes^44–46^. Incomplete X chromosome inactivation is associated with sex differences in gene expression and phenotype^46,47^. These sex differences are often detected in multiple tissues and can contribute to sex differences in disease risk, symptom profile, and severity. While we performed the *APOE ε4* conditional analysis in our cohort, we did not perform this analysis with other autosomal risk variants as they have lower effect sizes, and we were underpowered. These analyses should be pursued in future work including a larger cohort with additional fine mapping and validation efforts.

Our cohort included both dementia with Lewy bodies and Parkinson’s disease dementia cases. Although these two dementias may have differences in the progression rates, and underlying pathophysiological mechanisms, there is substantial overlap^1^. Currently, dementia with Lewy bodies and Parkinson’s disease dementia can only be differentiated by the interval between the onset time of dementia and parkinsonism. This information is based on the patient or the caregiver’s report, is prone to recall bias, and is often unavailable retrospectively. Additionally, the most up-to-date Parkinson’s disease diagnostic criteria no longer exclude dementia^48^, and the research criteria for the prodromal stages of Parkinson’s disease and dementia with Lewy bodies overlap^24,49^. Thus, due to the clinical similarities alongside difficulties in recruiting this group of individuals, studies may focus on LBD, the umbrella term, instead, and we have followed this approach^50^. Clinical profiles of dementia with Lewy bodies and Alzheimer’s can also overlap, with higher rates of misdiagnosis for females^1,5^. Genetic analyses with autosomal variants implicate that risk profiles and pathways in LBD overlap with Alzheimer’s and Parkinson’s diseases^7^. As Alzheimer’s pathology is frequent in people with LBD, detailed pathological and clinical data of the case and control cohorts can be helpful to better describe and analyze the genetic associations with individual clinical features taking into account the underlying pathologies.

The clinical diagnostic accuracy in LBD is limited due to common co-pathologies, and most of the available data in the field stem from those with a typical presentation of LBD^51^. Including LBD participants with a typical presentation might have only provided a partial insight into pathophysiology. In addition, females are more commonly misdiagnosed, and the currently available data may not accurately represent females with Lewy body pathology^4,5,33^. The future clinical implementation of alpha-synuclein and other co-pathology biomarkers may help identify more individuals with Lewy body pathology within the community.

Another limitation of our study was its focus on individuals with European ancestry. Genetic associations for neurodegenerative diseases differ across ethnic and racial groups^52^, and female sex has been associated with a higher LBD risk in people identifying as African American or Hispanic^53^. The future availability of diverse cohorts of people affected by Lewy body disorders, together with new frameworks for analyzing admixed populations^17^, will allow a better understanding of the genetic underpinnings of this disease spectrum.

In summary, our findings complement previously reported autosomal genetic risk factors for LBD and support the role of genetic factors as important drivers for sex differences in LBD. Studies with more extensive and well-defined cohorts are needed to replicate and determine the generalizability of our findings. We have made the individual-level whole-genome sequence data publicly available to facilitate such future efforts. We have also made the analysis pipeline needed to perform the XWAS analysis freely accessible to facilitate sex chromosome analysis in other diseases (https://github.com/ruthchia/XWAS_LBD).

## Methods

### Participants

Analyses were conducted in a cohort of 2591 LBD cases and 4391 neurologically healthy controls of European ancestry^7^. Detailed information about the cohort has been published elsewhere^7^. The LBD cases included 948 females and 1643 males obtained from seventeen European and North American sites and consortia. Within the LBD group, 1789 patients were autopsy-confirmed, and 802 had clinically probable LBD.

The control subjects comprised 2427 females and 1964 males without cognitive decline or neurological disorders based on history and neurological examination. Of these control participants, 605 had neuropathological data confirming a lack of neurodegenerative diseases. Local institutional review boards of participating institutions approved the study. All participants or their legally authorized representatives signed informed consents, and the study was carried out in accordance with the Declaration of Helsinki.

### Quality control

**Fig. 3:**
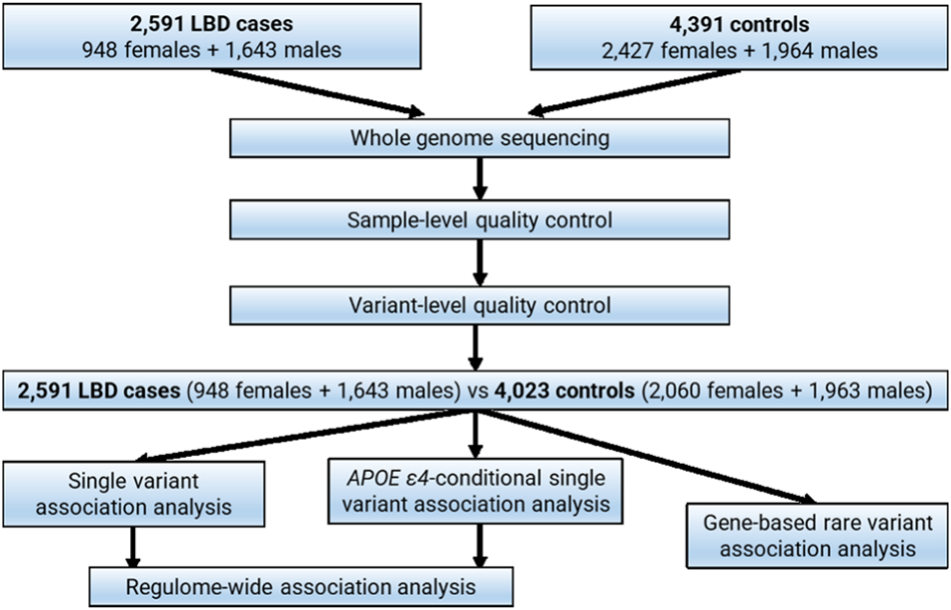
Study workflow diagram showing the LBD cases and neurologically healthy controls included in the X chromosome quality control and the final number of samples in the X-wide association and rare variant gene-based analysis.

The study workflow is summarized in Fig. 3. All study participants underwent whole-genome sequencing using PCR-free, 150-base-pair, paired-end sequencing on an Illumina Hi-Seq X-Ten platform^7^. The average coverage per genome was 35x. Sequence alignment to the reference genome (GRCH38DH) and variant calling followed the GATK Best Practices, as described elsewhere^7,54^. The individual-level data are publicly available at dbGaP (accession number phs001963) and on the AMP PD web portal (https://amp-pd.org/).

Sample-level quality control checks were consistent with those reported in our original study of autosomal variants^7^. Briefly, genomes were excluded for the following reasons: (1) high contamination rate (>5% based on the VerifyBamID freemix metric); (2) excessive heterozygosity rate (exceeding ±0.15 F-statistic); (3) low call rate (≤95%); (4) discordance between reported sex and genotypic sex; (5) duplicate samples (determined by pi-hat statistics >0.8); (6) non-European ancestry based on principal components analysis when compared to the HapMap 3 Genome Reference Panel; and (7) samples that were closely related to each other (defined by having pi-hat >0.125, one member of each pair was removed).

Next, we performed X chromosome-specific variant-level quality control checks for males and females separately according to standard guidelines^13^. We excluded: (1) variants with non-random missingness between cases and controls (*p*-value ≤ 1.0 × 10^−4^); (2) variants with haplotype-based non-random missingness (*p*-value ≤ 1.0 × 10^−4^); and (3) variants with an overall missingness rate of ≥2%. We then merged the remaining male and female data and excluded: (1) variants with >5% difference in MAF between male and female controls; (2) variants with >5% difference in missingness rates between male and female controls; (3) variants in the PARs (chrX:10001–2781479 for PAR1 region, and chrX:155701383–156030895 for PAR2 region) and (4) variants that failed the Hardy–Weinberg equilibrium exact test (*p*-value < 1.0 × 10^−4^) in female controls.

After these quality control steps, 2591 LBD cases (*n* = 948 females, *n* = 1643 males) and 4023 controls (*n* = 2060 females, *n* = 1963 males) were available. Only single nucleotide polymorphisms and small indels (<50bp compared to reference allele) were retained for the analysis.

### Haplotype block determination on autosomal and X chromosomes

The number of haploblocks was estimated using the linkage disequilibrium method according to Haploview’s interpretation of the haploblock definition^55^. Using PLINK (version 1.9), the following parameters were applied: *--hwe 1* *×* *10*^*−6*^ *midp --blocks-min-maf 0.01 --blocks-max-kb 1000*. These parameters partitioned the haplotype blocks to a maximum block size of 1 Mb using common variants with an MAF >1% that pass the Hardy–Weinberg equilibrium filter (*p*-value *>* 1 × 10^−6^). To cross-validate the estimated numbers across different datasets of European ancestry and the effect of variant density on haplotype block partition^56^, the analysis was performed on the LBD case-control cohort used in this study, a subset of TOPMed cohort (dbGAP Accession phs001662, phs000974, and phs000951; *n* = 6310 individuals) and The 1000 Genomes project (phase 3, *n* = 503 individuals, http://ftp.1000genomes.ebi.ac.uk/vol1/ftp/release/20130502/). Chromosomal lengths were obtained from NCBI (www.ncbi.nlm.nih.gov/grc/human/data).

### Single-variant association analyses

The association analysis was performed using PLINK (version 2.0; https://www.cog-genomics.org/plink/2.0/) by applying the *--xchr-model* parameter and setting it to 2, which is the default parameter. Due to the haploid status of the X chromosome in males, and diploid in females, this parameter setting codes males and females on the 0-2 scale (https://www.cog-genomics.org/plink/2.0/assoc#xchr_model). The *step* function (version 3.5.2; https://www.rdocumentation.org/packages/stats/versions/3.6.2/topics/step) in R was used to determine the minimum number of covariates required to correct for population substructure. To account for any population substructure driven by evolutionary differences between autosomes and X chromosome, the genetic principal components were generated from both autosomes and X chromosome for each sex-stratified and the overall cohort^12,13,15,57,58^. The *step* function uses the Akaike information criterion (AIC) in a stepwise algorithm to determine which covariates fits the model best^59^. The covariates from the model with the lowest AIC were selected for adjustment in the association analysis^50^. For the overall cohort (i.e., males and females combined), age, sex, and five principal components (PC1, PC3, PC4, PC5, PC7) were included as covariates in the regression analysis. For the sex-stratified analyses, age and four principal components (PC1, PC2, PC3, PC4) were adjusted in the female-only regression model. In comparison, age and five principal components (PC1, PC2, PC3, PC4, PC5) were included as covariates in the male-only regression model. This approach was chosen as the assumptions of uniform X-inactivation in females and a similar effect size between females and males are often invalid^46^. Using the R package “genpwr”, our study had 80% power to detect significant associations at an MAF ≥1% and an odds ratio >1.25 under the additive model.

For the *APOE ε4*-conditional analysis, the *APOE ε4* alleles were identified based on the genotypes at rs7412 and rs429358, and the dosages were assigned based on the number of *ε4* alleles carried per sample^19^. To perform the conditional analysis, the *APOE ε4* genotype was included as a covariate in the regression analysis described above.

### Regulome-wide association analysis (RWAS)

Enrichment analysis was performed to map risk variants to enhancers in a tissue-specific manner. Summary statistics from single-variant association analysis were used as input to identify candidate enhancers that may mediate disease risk by altering the regulation of nearby gene expression. The RWAS analysis employs MAGMA (version 1.10), where default parameters and framework were used^60^. Briefly, the analysis involved mapping cohort-specific genotypes to thirteen previously generated brain-related cell type-specific or tissue-specific regulatory features (https://data.nemoarchive.org/other/grant/sament/sament/RWAS)^60^. This was followed by association testing of each mapped regulatory feature and enhancer-set enrichment analysis. The brain enhancer maps were: cortex-derived primary cultured neurospheres (number of mapped enhancers = 811), ganglion eminence-derived primary cultured neurospheres (*n* = 1122), brain angular gyrus (*n* = 1842), brain anterior caudate (*n* = 1849), brain cingulate gyrus (*n* = 1852), brain germinal matrix (*n* = 930), brain hippocampus middle (*n* = 1528), brain inferior temporal lobe (*n* = 1818), brain dorsolateral prefrontal cortex (*n* = 1797), brain substantia nigra (*n* = 1791), fetal brain male (*n* = 934), fetal brain female (*n* = 934), and NH-A astrocytes primary cells (*n* = 1666). The Bonferroni significance threshold was set to 0.05/number of mapped enhancers per tested tissue. The GeneHancer database was used to identify candidate genes associated with significantly enriched enhancers^18^.

### Gene-based rare variant association analysis

To investigate the association of rare variants with an MAF <5%^61^, gene-level analysis was performed in RVTESTS (version 2.1.0) using the sequence kernel association test (SKAT)^62^. The variants on the X chromosome were annotated using default parameters in the Ensembl Variant Effect Predictor (version 101), followed by per gene aggregation of missense variants that had a MAF ≤5% and a MAC ≥ 3. The covariates used in the single variant analysis were applied to gene aggregation modeling. The threshold for significance in the gene burden analysis was set to be 8.31 × 10^−5^ (=0.05/602 X-chromosomal genes tested).

### Expression quantitative trait loci (eQTL) colocalization analysis

To investigate if any of the variants in risk regions were mediating risk via differential expression in brain tissues, we performed a colocalization analysis using the summary statistics from the female-only conditioned and unconditioned stratified XWAS analysis and eQTL association summary statistics from GTEx (version 8, https://gtexportal.org/) from the thirteen brain tissue types (amygdala, anterior cingulate cortex BA24, caudate basal ganglia, cerebellar hemisphere, cerebellum, cortex, frontal cortex BA9, hippocampus, hypothalamus, nucleus accumbens basal ganglia, putamen basal ganglia, spinal cord cervical c-1, and substantia nigra). Coloc (version 5.2.3; https://chr1swallace.github.io/coloc/articles/a03_enumeration.html) utilizes a Bayesian statistical framework that computes posterior probabilities to evaluate the probability of LBD loci and QTL sharing a single causal variant for each region. The index variant is the variant with the smallest *p*-value in each risk locus listed in Table 1. We evaluated the variants within the 1Mb region, flanking the index variant. Five hypotheses were tested: there is no association with either trait (hypothesis 0, H_0_); an associated LBD variant exists but no associated eQTL variant (H_1_); there is an associated eQTL variant but no associated LBD variant (H_2_); there is an association with an eQTL and LBD risk variant, but they are two independent variants (H_3_); and there is a shared associated LBD variant and eQTL variant within the analyzed region (H_4_). Default priors (*p*_1_ = 10^−4^ and *p*_2_ = 10^−4^, while prior *p*_12_ was set to *p*_12_ = 5 × 10^−6^) were used for the analysis. An XWAS locus was considered to colocalize with the region’s eQTL when the posterior probability of H_4_ was ≥ 0.70.

### Reporting summary

Further information on research design is available in the Nature Research Reporting Summary linked to this article.

### Supplementary information

Supplemental Material

Reporting Summary

## Data Availability

Individual-level whole-genome sequence data are freely accessible at https://github.com/ruthchia/XWAS_LBD. The genetic data are deposited in dbGAP – controlled access can be requested through the repository. The dbGAP study accession # is phs001963.v1.p1.

## Code availability

The analysis pipeline to perform the XWAS is freely accessible at https://github.com/ruthchia/XWAS_LBD.

## References

[1] Armstrong MJ (2019). Lewy Body Dementias. Continuum.

[2] Savica R (2013). Incidence of dementia with Lewy bodies and Parkinson disease dementia. JAMA Neurol..

[3] Nelson PT (2010). Association between male gender and cortical Lewy body pathology in large autopsy series. J. Neurol..

[4] Barnes LL, Lamar M, Schneider JA (2019). Sex differences in mixed neuropathologies in community-dwelling older adults. Brain Res..

[5] Bayram E, Coughlin DG, Banks SJ, Litvan I (2021). Sex differences for phenotype in pathologically defined dementia with Lewy bodies. J. Neurol. Neurosurg. Psychiatry.

[6] Sanghvi H, Singh R, Morrin H, Rajkumar AP (2020). Systematic review of genetic association studies in people with Lewy body dementia. Int J. Geriatr. Psychiatry.

[7] Chia R (2021). Genome sequencing analysis identifies new loci associated with Lewy body dementia and provides insights into its genetic architecture. Nat. Genet..

[8] Gibbons, E. et al. Identification of a sex-specific genetic signature in dementia with Lewy bodies: a meta-analysis of genome-wide association studies. *medRxiv*, 10.1101/2022.11.22.22282597 (2022).

[9] Gottipati S, Arbiza L, Siepel A, Clark AG, Keinan A (2011). Analyses of X-linked and autosomal genetic variation in population-scale whole genome sequencing. Nat. Genet.

[10] Tapper W (2005). A map of the human genome in linkage disequilibrium units. Proc. Natl Acad. Sci. USA.

[11] Skov L (2023). Extraordinary selection on the human X chromosome associated with archaic admixture. Cell Genomics.

[12] Gao F (2015). XWAS: A Software Toolset for Genetic Data Analysis and Association Studies of the X Chromosome. J. Heredity.

[13] Chang D (2014). Accounting for eXentricities: Analysis of the X Chromosome in GWAS Reveals X-Linked Genes Implicated in Autoimmune Diseases. PLoS One.

[14] Gomes I (2020). Twenty Years Later: A Comprehensive Review of the X Chromosome Use in Forensic Genetics. Front. Genet..

[15] Le Guen Y (2021). Common X-Chromosome Variants Are Associated with Parkinson Disease Risk. Ann. Neurol..

[16] Napolioni V, Khan RR, Greicius MD (2017). Chromosome X-wide association study identifies a new locus for late-onset Alzheimer’s disease on XQ25. Alzheimers Dement..

[17] Leal TP (2023). X‐Chromosome Association Study in Latin American Cohorts Identifies New Loci in Parkinson’s Disease. Mov. Disord..

[18] Fishilevich S (2017). GeneHancer: genome-wide integration of enhancers and target genes in GeneCards. Database.

[19] Kaivola, K., Shah, Z., Chia, R. & Scholz, S. W. Genetic evaluation of dementia with Lewy bodies implicates distinct disease subgroups. *Brain* awab402, 10.1093/brain/awab402 (2021). 10.1093/brain/awab402 PMC9423712 35381062

[20] Kim EK, Choi E-J (2010). Pathological roles of MAPK signaling pathways in human diseases. Biochim. Biophys. Acta.

[21] Nguyen K (2022). MAP3K Family Review and Correlations with Patient Survival Outcomes in Various Cancer Types. Front. Biosci..

[22] Backman JD (2021). Exome sequencing and analysis of 454,787 UK Biobank participants. Nature.

[23] Nag A (2022). Human genetics uncovers MAP3K15 as an obesity-independent therapeutic target for diabetes. Sci. Adv..

[24] Heinzel S (2019). Update of the MDS research criteria for prodromal Parkinson’s disease. Mov. Disord..

[25] Zhang J (2017). An updated meta-analysis of cohort studies: Diabetes and risk of Alzheimer’s disease. Diabetes Res Clin. Pr..

[26] Wang T (2020). Vascular, inflammatory and metabolic risk factors in relation to dementia in Parkinson’s disease patients with type 2 diabetes mellitus. Aging.

[27] Boot BP (2013). Risk factors for dementia with Lewy bodies: A case-control study. Neurology.

[28] Sundermann EE (2021). Prediabetes Is Associated With Brain Hypometabolism and Cognitive Decline in a Sex-Dependent Manner: A Longitudinal Study of Nondemented Older Adults. Front Neurol..

[29] Hardy J, de Strooper B, Escott-Price V (2022). Diabetes and Alzheimer’s disease: shared genetic susceptibility?. Lancet Neurol..

[30] Salomon-Zimri S (2019). The Role of MAPK’s Signaling in Mediating ApoE4-Driven Pathology In Vivo. Curr. Alzheimer Res.

[31] Placanica L, Zhu L, Li Y-M (2009). Gender- and Age-Dependent γ-Secretase Activity in Mouse Brain and Its Implication in Sporadic Alzheimer Disease. PLoS One.

[32] Oikawa N, Ogino K, Masumoto T, Yamaguchi H, Yanagisawa K (2010). Gender effect on the accumulation of hyperphosphorylated tau in the brain of locus-ceruleus-injured APP-transgenic mouse. Neurosci. Lett..

[33] Bayram E, Coughlin DG, Litvan I (2022). Sex Differences for Clinical Correlates of Alzheimer’s Pathology in People with Lewy Body Pathology. Mov. Disord..

[34] Lopez-Lee C, Kodama L, Gan L (2022). Sex Differences in Neurodegeneration: The Role of the Immune System in Humans. Biol. Psychiatry.

[35] López-Cerdán A (2022). Unveiling sex-based differences in Parkinson’s disease: a comprehensive meta-analysis of transcriptomic studies. Biol. Sex. Differ..

[36] Hansen DV (2013). Non-epithelial stem cells and cortical interneuron production in the human ganglionic eminences. Nat. Neurosci..

[37] Liu Y (2013). Medial ganglionic eminence–like cells derived from human embryonic stem cells correct learning and memory deficits. Nat. Biotechnol..

[38] Jellinger KA (2015). The cholinergic basal forebrain in Lewy body dementia and Alzheimer’s disease. J. Neurol..

[39] Li Y (2021). Tex13a Optimizes Sperm Motility via Its Potential Roles in mRNA Turnover. Front Cell Dev. Biol..

[40] Matos B, Publicover SJ, Castro LFC, Esteves PJ, Fardilha M (2021). Brain and testis: more alike than previously thought?. Open Biol..

[41] Royer‐Bertrand B (2021). CNOT2 haploinsufficiency in a 40‐year‐old man with intellectual disability, autism, and seizures. Am. J. Med Genet A.

[42] Martin R (2019). De novo variants in CNOT3 cause a variable neurodevelopmental disorder. Eur. J. Hum. Genet..

[43] Sun L-L, Yang S-L, Sun H, Li W-D, Duan S-R (2019). Molecular differences in Alzheimer’s disease between male and female patients determined by integrative network analysis. J. Cell Mol. Med..

[44] Cotton AM (2013). Analysis of expressed SNPs identifies variable extents of expression from the human inactive X chromosome. Genome Biol..

[45] Carrel L, Willard HF (2005). X-inactivation profile reveals extensive variability in X-linked gene expression in females. Nature.

[46] Tukiainen T (2017). Landscape of X chromosome inactivation across human tissues. Nature.

[47] Deng X, Berletch JB, Nguyen DK, Disteche CM (2014). X chromosome regulation: diverse patterns in development, tissues and disease. Nat. Rev. Genet..

[48] Postuma RB (2015). MDS clinical diagnostic criteria for Parkinson’s disease. Mov. Disord..

[49] McKeith IG (2020). Research criteria for the diagnosis of prodromal dementia with Lewy bodies. Neurology.

[50] Weintraub D (2023). What’s in a Name? The Time Has Come to Unify Parkinson’s Disease and Dementia with Lewy Bodies. Mov. Disord..

[51] Coughlin DG, Hurtig HI, Irwin DJ (2019). Pathological Influences on Clinical Heterogeneity in Lewy Body Diseases. Mov. Disord..

[52] Chin AL, Negash S, Hamilton R (2011). Diversity and Disparity in Dementia: The Impact of Ethnoracial Differences in Alzheimer’s Disease. Alzheimer Dis. Assoc. Disord..

[53] Kurasz AM, Smith GE, McFarland MG, Armstrong MJ, O’Bryant S (2020). Ethnoracial Differences in Lewy Body Diseases with Cognitive Impairment. J. Alzheimers Dis..

[54] *Best Practices for Variant Calling with the GATK | Broad Institute*. https://www.broadinstitute.org/partnerships/education/broade/best-practices-variant-calling-gatk-1 (2015).

[55] Gabriel SB (2002). The Structure of Haplotype Blocks in the Human Genome. Science.

[56] Kim SA, Yoo YJ (2016). Effects of Single Nucleotide Polymorphism Marker Density on Haplotype Block Partition. Genomics Inf..

[57] Hernangomez-Laderas A (2023). Sex bias in celiac disease: XWAS and monocyte eQTLs in women identify TMEM187 as a functional candidate gene. Biol. Sex. Differ..

[58] Grunin, M. et al. Identifying X-Chromosome Variants Associated with Age-Related Macular Degeneration. *medRxiv*10.1101/2023.08.28.23294688 (2023). 10.1093/hmg/ddae141 PMC11630753 39324238

[59] Forster, M. & Sober, E. Aic Scores as Evidence. In *Philosophy of Statistics* 535–549 (Elsevier, 2011). 10.1016/B978-0-444-51862-0.50016-2.

[60] Casella AM, Colantuoni C, Ament SA (2022). Identifying enhancer properties associated with genetic risk for complex traits using regulome-wide association studies. PLoS Comput. Biol..

[61] Ma C, Boehnke M, Lee S (2015). Evaluating the Calibration and Power of Three Gene-Based Association Tests of Rare Variants for the X Chromosome. Genet. Epidemiol..

[62] Lee S (2012). Optimal Unified Approach for Rare-Variant Association Testing with Application to Small-Sample Case-Control Whole-Exome Sequencing Studies. Am. J. Hum. Genet..

